# Modulation of the lncRNA TCONS_00265853-miR-421-5p-CLOCK axis by Ziyin Buyang Formula in polycystic ovarian syndrome with circadian rhythm disruption: an integrated bioinformatics and experimental approach

**DOI:** 10.3389/fgene.2025.1658812

**Published:** 2025-10-08

**Authors:** Haixia Huang, Ling Sun, Fan Jia, Yong Tan, Yahong Zhou

**Affiliations:** ^1^ Department of Reproductive Medicine, Wuxi Affiliated Hospital of Nanjing University of Chinese Medicine, Wuxi, Jiangsu, China; ^2^ Institute of Traditional Chinese Medicine, Wuxi Affiliated Hospital of Nanjing University of Chinese Medicine, Wuxi, Jiangsu, China; ^3^ First Clinical Medical College, Nanjing University of Chinese Medicine, Nanjing, Jiangsu, China

**Keywords:** polycystic ovary syndrome, circadian rhythm disruption, Ziyin Buyang Formula, network pharmacology, traditional Chinese medicine, MAPK signaling pathway

## Abstract

**Background:**

Polycystic ovarian syndrome (PCOS), a common endocrine disorder in reproductive-aged women, is linked to circadian rhythm disruption, but the molecular mechanisms remain unclear. Dysregulation of the lncRNA TCONS_00265853-miR-421-5p-CLOCK axis may play an important role. Ziyin Buyang Formula (ZYBYF), a Traditional Chinese Medicine (TCM) targeting kidney Yin-Yang balance, shows therapeutic potential, though its mechanism is unclear. This study explores ZYBYF’s role in modulating this axis to improve circadian-disrupted PCOS.

**Methods:**

Network pharmacology identified ZYBYF’s bioactive components and targets, integrated with PCOS-associated genes and circadian disruption-related differentially expressed genes (DEGs) from ovarian tissues. A continuous light-induced PCOS rat model (10-week 24-h light exposure) was employed to validate ZYBYF’s effects. Interventions included ZYBYF treatment.

**Results:**

Bioinformatics identified 25 intersection targets, with MAPK signaling as the central pathway. *In vivo*, circadian disruption downregulated lncRNA TCONS_00265853 and *CLOCK*, elevated miR-421-5p, and hyperactivated MAPK (p-p38, p-ERK1/2, p-JNK), exacerbating ovarian apoptosis (↑BAX, ↓Bcl-2, p < 0.001). ZYBYF restored circadian axis components (↑TCONS_00265853, *CLOCK*; ↓miR-421-5p), suppressed MAPK activation, and normalized ovarian morphology and hormonal profiles (↓LH, T, ACTH; ↑E2, FSH, p < 0.001). PRKCA expression, a MAPK regulator, was rescued by ZYBYF, counteracting dysregulation of *IL1B*, *VEGFA*, and *TGFB1*.

**Conclusion:**

Circadian disruption exacerbates PCOS via the TCONS_00265853-miR-421-5p-*CLOCK* axis, driving MAPK hyperactivation and ovarian apoptosis. ZYBYF reverses these effects, restoring hormonal balance and follicular dynamics. This study provides mechanistic validation of ZYBYF’s efficacy, positioning it as a promising therapeutic strategy for circadian-disrupted PCOS. These findings suggest that ZYBYF may modulate circadian rhythm–related pathways in PCOS via the lncRNA–miRNA–CLOCK axis, warranting further mechanistic validation.

## Introduction

Polycystic ovary syndrome (PCOS) is a prevalent gynecological and endocrine disorder affecting women of reproductive age, with an incidence ranging from 5% to 18% (Azziz, 2016). Recognized as a leading cause of ovulatory infertility, PCOS is characterized by hyperandrogenemia and disrupted follicular development (American College of, 2018). The etiology of PCOS is multifaceted, governed by genetic, epigenetic, lifestyle, and environmental factors, yet remains incompletely understood (American College of, 2018).

Circadian rhythms play a crucial role in human growth and development (Moreno et al., 2019), reproduction (Mills and Kuohung, 2019; Peterlin et al., 2019), aging (Acosta-Rodriguez et al., 2021), and other physiological processes. Specifically, clock genes are implicated in various reproductive functions in women, including follicle maturation (Wiggins and Legge, 2016), ovulation (Silva et al., 2020), fertilization (Xu et al., 2016), embryo implantation (Chuffa et al., 2020), and parturition (Olcese et al., 2013). Disruptions in circadian rhythms have been associated with menstrual disorders (Attarchi et al., 2013), infertility (Sciarra et al., 2020), and miscarriage (Stocker et al., 2021). Aberrant expression of clock genes in PCOS suggests a potential link between circadian rhythm disturbances and the syndrome (Wang F. et al., 2021). However, the mechanisms by which circadian rhythm disruptions contribute to PCOS remain largely exploratory.

Emerging evidence indicates that long noncoding RNAs (lncRNAs) and microRNAs (miRNAs) are pivotal in regulating circadian rhythms and PCOS (Zhu and Belden, 2020). Our selection of the lncRNA TCONS_00265853–miR-421-5p–*CLOCK* axis is based on our prior transcriptomic data showing that, among many dysregulated non-coding RNAs in circadian-disrupted ovarian tissues, TCONS_00265853 was one of the most significantly downregulated transcripts predicted to bind miR-421-5p. Furthermore, miR-421-5p levels were significantly elevated and known to target *CLOCK* (Luo, 2021). This strong, reciprocal expression pattern led us to prioritize this specific axis for mechanistic investigation. The downstream regulatory mechanisms of this signaling axis, however, remain obscure.

According to Traditional Chinese Medicine (TCM) theory, the kidney is integral to reproductive health, crucially influencing the development and maturation of reproductive organs and ensuring robust reproductive function. This study is based on the therapeutic principles developed by Professor Xia Guicheng, a master of TCM, who integrated traditional gynecology with modern reproductive endocrinology (Fu et al., 2021). This integration led to the concept of cycle-based therapy: nourishing kidney yin during the follicular phase and tonifying kidney yang during the luteal phase. Consequently, the Ziyin formula (ZYF) and Buyang formula (BYF) were developed to balance this yin-yang dynamic and restore ovarian function. This combination, Ziyin Buyang Formula (ZYBYF), has shown significant clinical efficacy in managing PCOS (Zhou et al., 2016). Unlike previous studies that have broadly linked clock gene dysregulation to PCOS, this work specifically investigates a novel lncRNA-miRNA regulatory axis and explores the therapeutic mechanism of ZYBYF through an integrated bioinformatic and experimental approach.

This study investigates the downstream regulatory mechanisms of the lncRNA TCONS_00265853-miR-421-5p-*CLOCK* axis in PCOS associated with circadian rhythm disruption, and elucidates the intervention mechanism of ZYBYF. Initial gene identification was conducted through bioinformatics analyses, followed by validation in rat models of PCOS with circadian rhythm disruption. The findings aim to enhance our understanding of PCOS and contribute to more effective management strategies.

## Materials and methods

### Screening of the potential targets of ZYBYF

The Ziyin Buyang Formula (ZYBYF) comprises two distinct formulae: the Ziyin Formula (ZYF) and the Buyang Formula (BYF), with detailed compositions provided in Table 1. Bioactive components of ZYBYF constituents were retrieved from the Traditional Chinese Medicine Systems Pharmacology Database (TCMSP, https://ibts.hkbu.edu.hk/LSP/tcmsp.php) and supplementary literature (Li et al., 2021). Active compounds were selected based on pharmacokinetic criteria: oral bioavailability (OB) ≥30% and drug-likeness (DL) ≥0.18. Potential targets were predicted using TCMSP algorithms, with gene symbols standardized via the UniProtKB database (https://www.uniprot.org).

**TABLE 1:** Composition of Ziyin Buyang Formula (ZYBYF).

Formula	Herbal component	Pinyin name	Latin name	Dosage (g) in decoction
Ziyin Formula (ZYF)	Angelica sinensis (Oliv.) Diels	Danggui	*Angelicae Sinensis Radix*	10
	Paeonia lactiflora Pall.	Baishao	*Paeoniae Radix Alba*	12
	Rehmannia glutinosa (Gaertn.) DC.	Dihuang	*Rehmanniae Radix Praeparata*	15
	Cornus officinalis Siebold & Zucc.	Shanzhuyu	*Corni Fructus*	12
	Cuscuta chinensis Lam.	Tusizi	*Cuscutae Semen*	15
Buyang Formula (BYF)	Dipsacus asper Wall. ex DC.	Xuduan	*Dipsaci Radix*	12
	Dioscorea oppositifolia L.	Shanyao	*Dioscoreae Rhizoma*	15
	Gynochthodes officinalis (F.C. How) Razafim. & B. Bremer	Bajitian	*Morindae Officinalis Radix*	10
	Epimedium sagittatum (Siebold & Zucc.) Maxim.	Yinyanghuo	*Epimedii Herba*	10
	Cullen corylifolium (L.) Medik	Buguzhi	*Psoraleae Fructus*	10

### Targets of PCOS

PCOS-associated targets were systematically collated from four databases: GeneCards (https://www.genecards.org/; keyword “Polycystic ovary syndrome”, relevance score >20), DrugBank (https://go.drugbank.com/), DisGeNET (https://www.disgenet.org/), and PharmGKB (https://www.pharmgkb.org/). Duplicate entries were removed to generate a consolidated PCOS target repository.

### Targets of circadian rhythm disruption in ovarian tissue

Differentially expressed genes (DEGs) from ovarian tissues of circadian-disrupted rats versus controls were identified through transcriptomic analysis. DEGs were defined by an adjusted p-value <0.05 and an absolute log2-fold change >1.0. Orthologous human gene mapping was performed using the NCBI HomoloGene database (https://www.ncbi.nlm.nih.gov/homologene) to translate rat gene symbols into their human equivalents. The mapping was followed by manual verification to resolve ambiguities, ensuring functional conservation by cross-referencing gene annotations.

### Integrated therapeutic target identification

Venn diagram analysis (BioVenn web tool) identified overlapping targets among three datasets: ZYBYF component targets (1,258 genes), PCOS-associated targets (892 genes), and circadian disruption-related DEGs (327 genes). The intersection (68 genes) was retained for subsequent analyses.

### Functional enrichment analysis

Gene Ontology (GO) and Kyoto Encyclopedia of Genes and Genomes (KEGG) pathway enrichment analyses were conducted using the R package clusterProfiler (v4.0). Statistical significance was determined at adjusted p < 0.05 and q-value <0.05. Protein-protein interaction networks were constructed using STRING (v11.5; confidence score >0.7) and visualized in Cytoscape (v3.9.1).

### Animal model development

Thirty specific pathogen-free (SPF) female Sprague-Dawley rats (7 weeks old, 180–220 g) were obtained from Beijing Vital River Laboratory Animal Technology Co., Ltd. (SCXK 2021-0011). Animals were housed under controlled conditions (22°C ± 1 °C, 50%–60% humidity) with *ad libitum* access to food and water. Following 1-week acclimatization under 12:12 light-dark (LD) cycles, rats were randomized into three groups (n = 10/group):

• Control (D/L): Standard LD cycles
• Model (L/L): Continuous light exposure (LL)
• ZYBYF group: LL + ZYBYF treatment

Circadian disruption was induced via 24-h light exposure (500 ± 20 lux intensity, LED panels) for 10 weeks (Ma et al., 2018). Drug administration commenced post-induction: ZYF (5.88 mg/kg) during proestrus/estrus phases and BYF (6.195 mg/kg) during metestrus/diestrus phases via oral gavage. All procedures were approved by the Nanjing University of Chinese Medicine Animal Ethics Committee (202201A002).

### Histopathological evaluation

Ovarian tissues were fixed in 4% paraformaldehyde (24 h), paraffin-embedded, and sectioned (5 μm thickness). Hematoxylin and eosin (H&E) staining was performed using standard protocols. Follicular morphology and cystic changes were assessed by two blinded pathologists using an Olympus BX53 microscope.

### Hormonal quantification

Serum anti-Müllerian hormone (AMH, #JM-01626R1), estradiol (E2, #JM-01981R1), luteinizing hormone (LH, #JM-02207R1), follicle-stimulating hormone (FSH, #JM-01972R1), testosterone (T, #JM-01983R1), and adrenocorticotropic hormone (ACTH, #JM-01971R1) levels were measured using ELISA kits (Jingmei Biotech) according to manufacturer protocols. Absorbance was quantified at 450 nm using a microplate reader (BioTek Synergy H1).

### Western blot analysis

Total protein was extracted using RIPA lysis buffer supplemented with protease inhibitors. Proteins (20 μg/lane) were separated on 10% SDS-PAGE gels and transferred to PVDF membranes. After blocking with 5% BSA, membranes were incubated overnight at 4 °C with primary antibodies: p-p38 (Abclonal AP1311, 1:2,000), p-ERK1/2 (Zen-Bio 310289, 1:1,000), p-JNK (Abclonal AP0631, 1:2,000), BAX (Proteintech 50599-2-Ig, 1:5,000), Bcl-2 (Proteintech 68103-1-Ig, 1:5,000), CLOCK (Abcam, ab229495, 1:1,000), β-actin (Zen-Bio 200068-8F10, 1:5,000). HRP-conjugated secondary antibodies (1:3,000) were detected using ECL Prime (Amersham). Band intensities were quantified via ImageJ (v1.53k).

### Quantitative reverse transcription PCR (RT-qPCR)

Total RNA was isolated using TRIzol reagent (Invitrogen) and reverse-transcribed with PrimeScript RT Master Mix (Takara). SYBR Green-based qPCR was performed on a StepOnePlus system (Applied Biosystems) using the following cycling parameters: 95 °C for 1 min; 40 cycles of 95 °C for 20 s, 60 °C for 20 s, and 72 °C for 30 s. Primer sequences: lncRNA TCONS_00265853: F: 5′-CCT​TCC​CTC​CTC​AAG​TTG​CCT-3′, R: 5′-GTC​CTC​ACA​TAG​ACA​ATG​CCA​AA-3'; miR-421-5p: F: 5′-CAC​ACA​GAA​GGC​CAC​AAA​AA-3′, R: 5′-TAT​GGT​TTT​GAC​GAC​TGT​GTG​AT-3'; *Clock*: F: 5′-TCT​CTT​CCA​AAC​CAG​ACG​CC-3′, R: 5′-TGC​GGC​ATA​CTG​GAT​GGA​AT-3'; *β-actin* (reference): F: 5′-AGG​GTG​TGA​TGG​TGG​GTA​TG-3′, R: 5′-AGG​ATG​CCT​CTC​TTG​CTC​TG-3'. Relative expression was calculated using the 2^−ΔΔCT^ method with normalization to *β-actin*.

### TUNEL staining

Apoptosis was assessed using a TUNEL assay kit (Solarbio T2130). Deparaffinized sections were incubated with proteinase K (20 μg/mL), equilibrated, and labeled with TdT reaction mix (37 °C, 1 h). Nuclei were counterstained with DAPI. Images were captured using a Nikon Eclipse CI fluorescence microscope (20× objective). Apoptotic indices were calculated as TUNEL-positive cells/total cells ×100% in five random fields per section.

### Statistical analysis

Data are expressed as mean ± SEM. Normality of data was assessed using the Kolmogorov-Smirnov test. For normally distributed data, one-way ANOVA with Newman-Keuls *post hoc* test was used. Non-parametric data were analyzed using the Kruskal-Wallis test with Dunn’s correction. For bioinformatics enrichment analysis, p-values were corrected for multiple comparisons using the Benjamini-Hochberg method. Statistical significance was defined as p < 0.05. All analyses were performed in SPSS 23.0 (IBM).

## Results

### Targets of ZYBYF in the treatment of PCOS with circadian rhythm disruption

**FIGURE 1:**
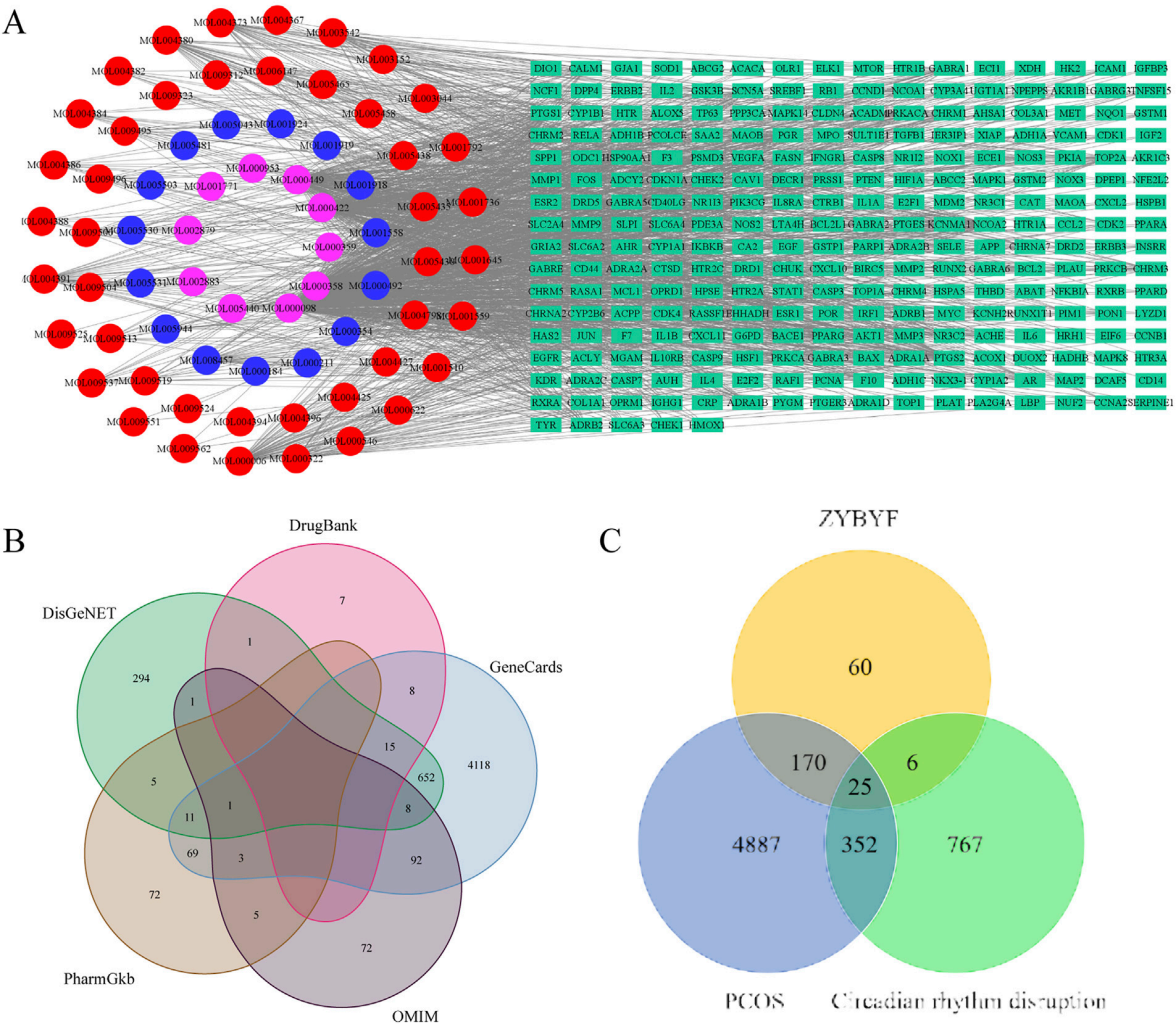
Targets of ZYBYF in the treatment of PCOS with circadian rhythm disruption. **(A)** A network illustrating the relationship between the active ingredients of Ziyin Buyang Formula (ZYBYF) and their protein targets. Circles represent active ingredients (blue for Ziyin Formula, ZYF; red for Buyang Formula, BYF; pink for common ingredients), and green rectangles represent protein targets. **(B)** Venn diagram showing the overlap of targets for Polycystic Ovary Syndrome (PCOS) from multiple databases. **(C)** Venn diagram showing the 25 intersection genes among ZYBYF targets, PCOS-related genes, and genes dysregulated by circadian rhythm disruption (CRD).

Through the identification of active ingredients, 40 compounds from ZYF and 66 from BYF were recognized. This led to the determination of 226 action targets for ZYF and 239 for BYF. After eliminating duplicates, a total of 261 unique ZYBYF action targets were identified (Figure 1A). Additionally, 5434 PCOS-associated targets were compiled from various databases: 4977 from GeneCards, 166 from PharmGKB, 988 from DisGeNET, 31 from DrugBank, and 182 from OMIM (Figure 1B). Moreover, 1,415 differential ovarian genes were identified in rats exposed to continuous light, with 1,150 human homologs obtained from the HomoloGene database. Analysis revealed 25 intersection genes among the 261 potential ZYBYF targets, the 5,434 PCOS targets, and the 1,150 circadian rhythm disruption targets, which represent the target genes of ZYBYF in treating PCOS with circadian rhythm disruption (Figure 1C).

### GO functional enrichment and KEGG pathway analysis

**FIGURE 2:**
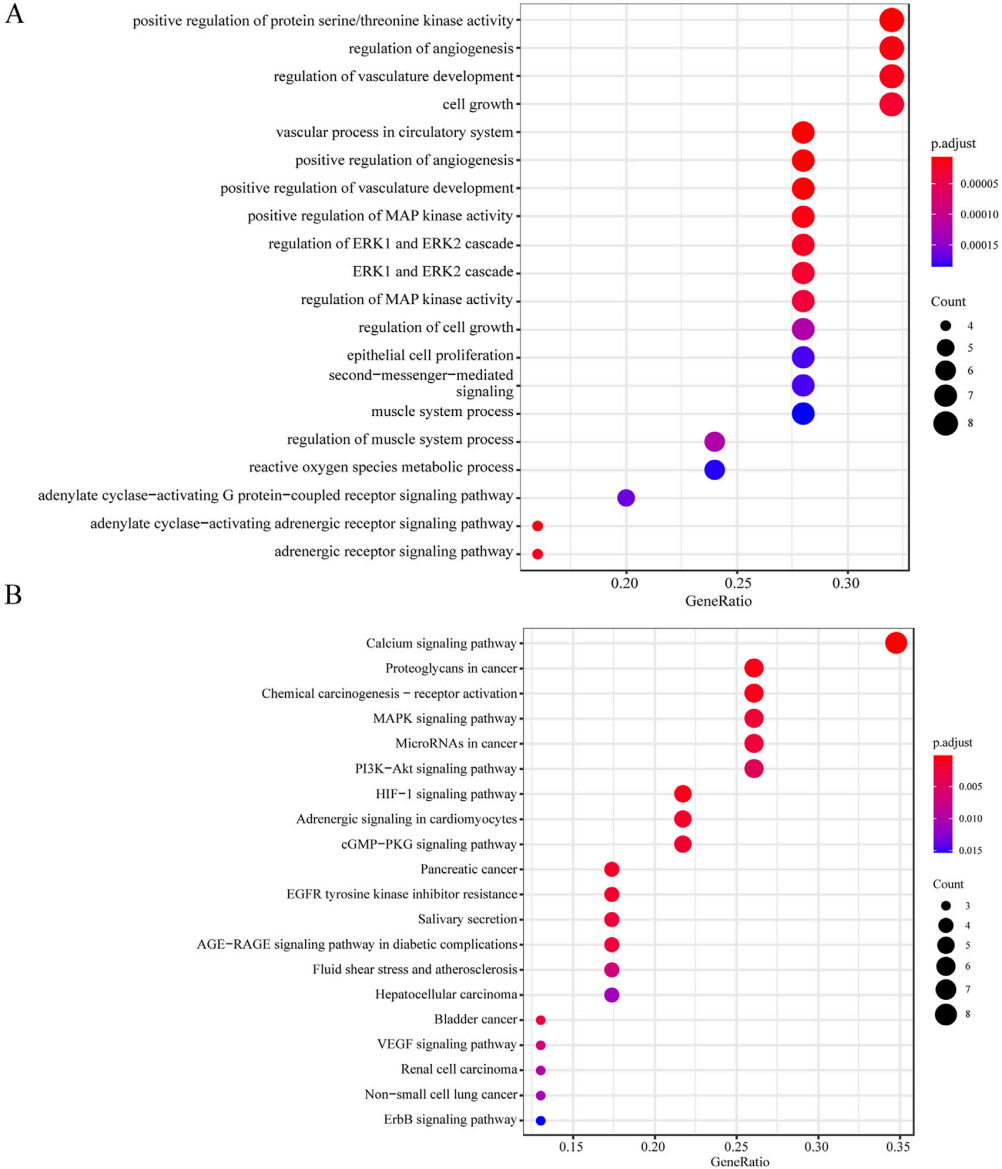
GO and KEGG enrichment analysis of intersection genes. **(A)** Top 20 enriched Gene Ontology (GO) biological process terms for the intersection genes. **(B)** Top 20 enriched Kyoto Encyclopedia of Genes and Genomes (KEGG) pathways. Dot size corresponds to the number of genes enriched, and color represents the adjusted p-value.

The 25 intersection genes underwent analysis using the R package, yielding 450 biological processes in GO functional enrichment and 38 pathways in KEGG pathway analysis. The 20 most significant terms are presented in Figure 2. The MAPK signaling pathway emerged as a pivotal pathway.

### Intersection target genes involved in the lncRNA-miRNA-mRNA regulatory axis

**FIGURE 3:**
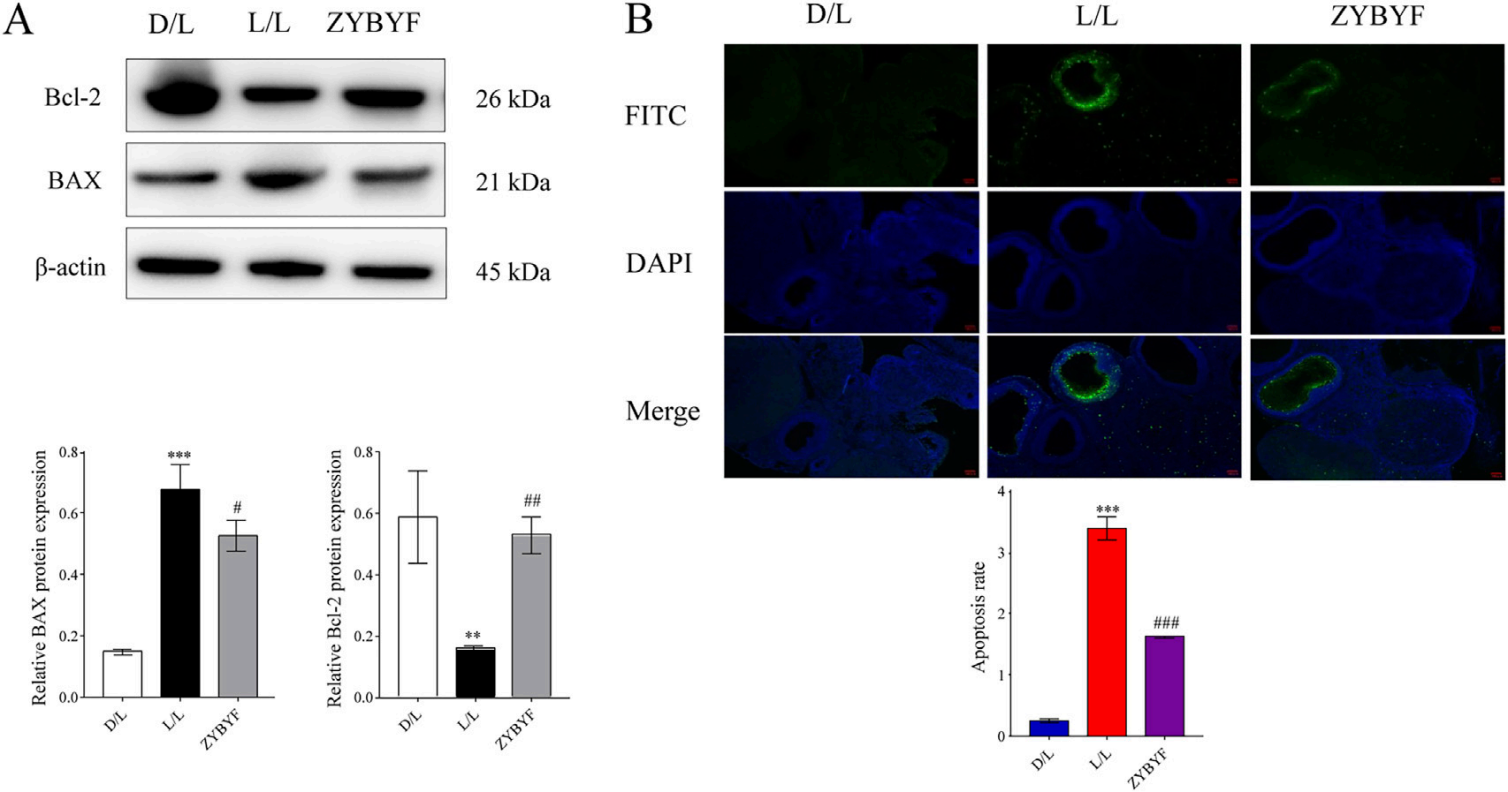
ZYBYF mitigates ovarian apoptosis in circadian-disrupted rats. **(A)** Protein expression of apoptosis-related markers BAX and Bcl-2 in ovarian tissues from the D/L (control), L/L (model), and ZYBYF groups, assessed by Western blot. β-actin was used as a loading control. **(B)** Quantification of ovarian cell apoptosis by TUNEL staining. Scale bar = 100 µm. Blue fluorescence indicates DAPI-stained nuclei, and green fluorescence indicates TUNEL-positive apoptotic cells. Data are presented as mean ± SEM (n = 10 per group). ***P < 0.001 vs. D/L group; ##P < 0.01, ###P < 0.001 vs. L/L group.

MAPK signaling is crucial in regulating circadian rhythm and is closely linked to PCOS (Dehghani et al., 2020). Enrichment analysis indicated that the MAPK signaling pathway is pivotal in PCOS with circadian rhythm disruption. STRING data predicted an interaction between CLOCK and intersection genes enriched in the MAPK signaling pathway (PRKCA, IL1B, ERBB2, KDR, VEGFA, and TGFB1), suggesting that CLOCK may target PRKCA to regulate the MAPK pathway (Figure 3).

### Effects of circadian rhythm disruption on the general condition of rats and the treatment results of ZYBYF

**FIGURE 4:**
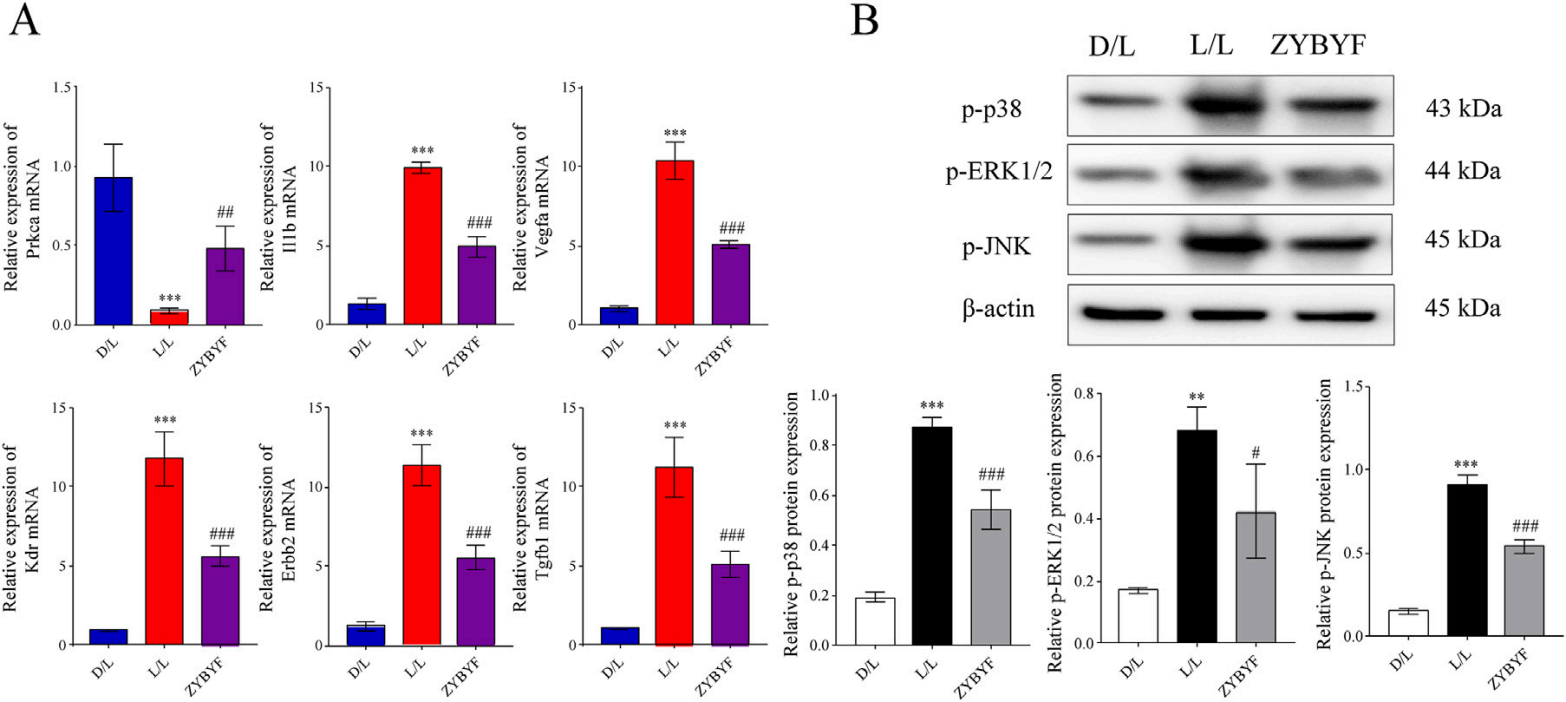
ZYBYF restores ovarian function by inhibiting the MAPK signaling pathway. **(A)** The mRNA expression of intersection genes enriched in the mitogen-activated protein kinase (MAPK) signaling pathway (*Prkca, Il1b, Vegfa, Kdr, Erbb2, Tgfb1*). **(B)** Protein expression of phosphorylated p38, ERK1/2, and JNK. The top panel shows representative Western blots, and the bottom panel shows densitometric quantification. Data are presented as mean ± SEM (n = 10 per group). **P < 0.01, ***P < 0.001 vs. D/L group; ##P < 0.01, ###P < 0.001 vs. L/L group.

Continuous light exposure for 10 weeks disrupted the estrous cycle in rats (Figure 4A), resulting in reduced body weights (Figures 4B,C), increased ovary weight and ovarian index (Figures 4D,E), and the appearance of polycystic ovarian morphology with increased cystic follicles and reduced granulosa cell layers (Figure 4F). Serum levels of AMH, LH, T, and ACTH increased, while E2 and FSH levels decreased (Figure 4G), confirming that circadian rhythm disruption induces PCOS in rats. Following ZYBYF treatment, the estrous cycle, body weights, ovarian morphology, and hormonal profiles were significantly restored (Figure 4).

### Expression of lncRNA TCONS_00265853, miR-421-5p, and *CLOCK* mRNA in rat ovarian tissue

**FIGURE 5:**
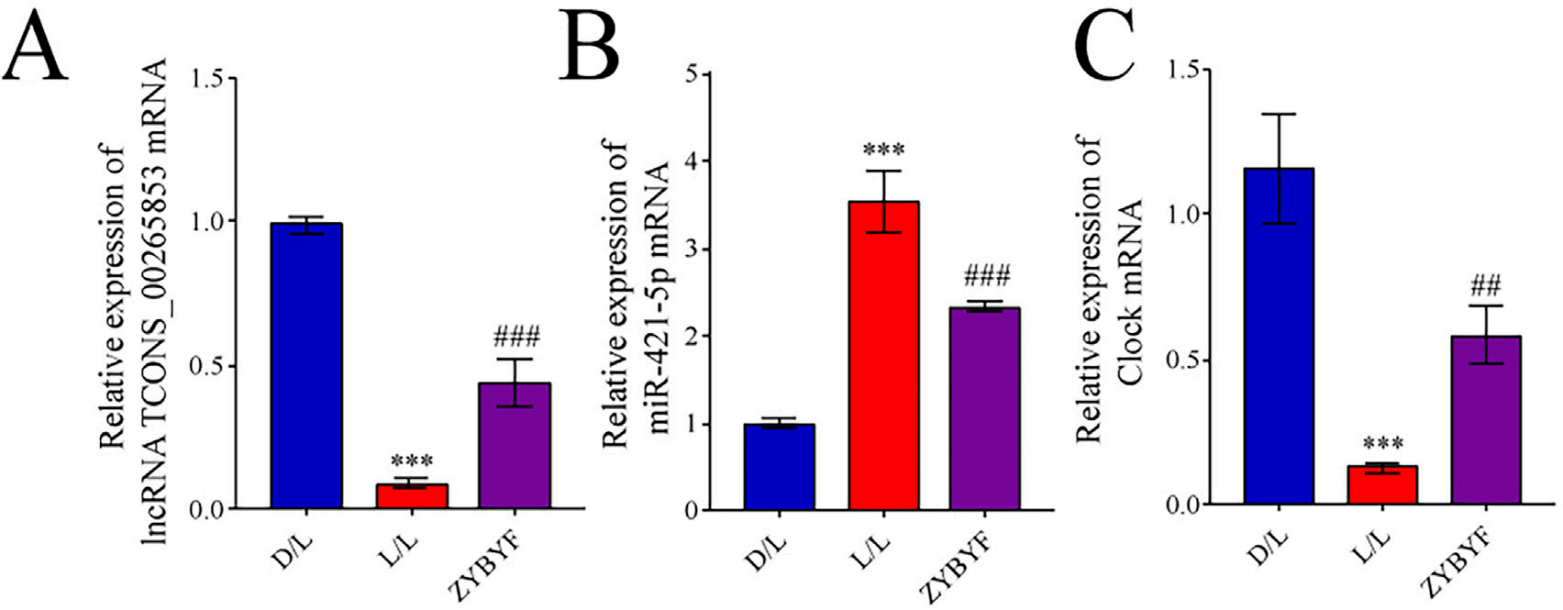
ZYBYF modulates the lncRNA TCONS_00265853-miR-421-5p-*CLOCK* axis in rat ovarian tissue. Relative mRNA expression of **(A)** lncRNA TCONS_00265853, **(B)** miR-421-5p, and **(C)** *CLOCK* in ovarian tissues. Data are presented as mean ± SEM (n = 10 per group). ***P < 0.001 vs. D/L group; ##P < 0.01, ###P < 0.001 vs. L/L group.

In rats exposed to continuous light, the expression of lncRNA TCONS_00265853 and *CLOCK* were downregulated, while miR-421-5p expression was upregulated (Figure 5). Post-ZYBYF treatment, the expressions of lncRNA TCONS_00265853 and *CLOCK* increased, and miR-421-5p expression decreased, suggesting ZYBYF treatment helps normalize this regulatory axis.

### ZYBYF improves ovarian function by inhibiting the MAPK signaling pathway

**FIGURE 6:**
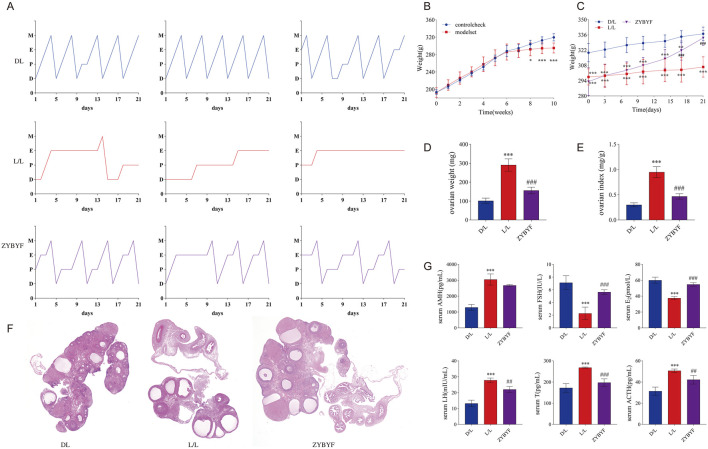
ZYBYF alleviates PCOS phenotypes in rats with circadian rhythm disruption. **(A)** Estrous cycle monitoring for 21 consecutive days. D (diestrus); P (proestrus); E (estrus); M (metestrus). **(B)** Body weight changes during the modeling period. **(C)** Body weight changes during the treatment period. **(D)** Ovary weight. **(E)** Ovarian index. **(F)** H&E staining of ovarian tissue. Scale bar = 1 mm. Blue arrow, developing follicle; Black arrow, corpus luteum; Red arrow, cystic follicle. **(G)** Serum hormone levels. Data are presented as mean ± SEM (n = 10 per group). ***P < 0.001 vs. D/L group; ##P < 0.01, ###P < 0.001 vs. L/L group.

In ovarian tissues of rats exposed to continuous light, *Prkca* mRNA expression decreased, while *Il1b, Vegfa, Kdr, Erbb2*, and *Tgfb1* expression increased (Figure 6A). At the protein level, the expression of p-p38, p-ERK1/2, and p-JNK increased, indicating MAPK pathway activation (Figure 6B). After ZYBYF treatment, *Prkca* expression was upregulated, while the expression of the other five genes was downregulated (Figure 6A). Concurrently, the phosphorylation levels of p-p38, p-ERK1/2, and p-JNK decreased (Figure 6B).

### ZYBYF mitigates ovarian apoptosis in circadian-disrupted rats

**FIGURE 7:**
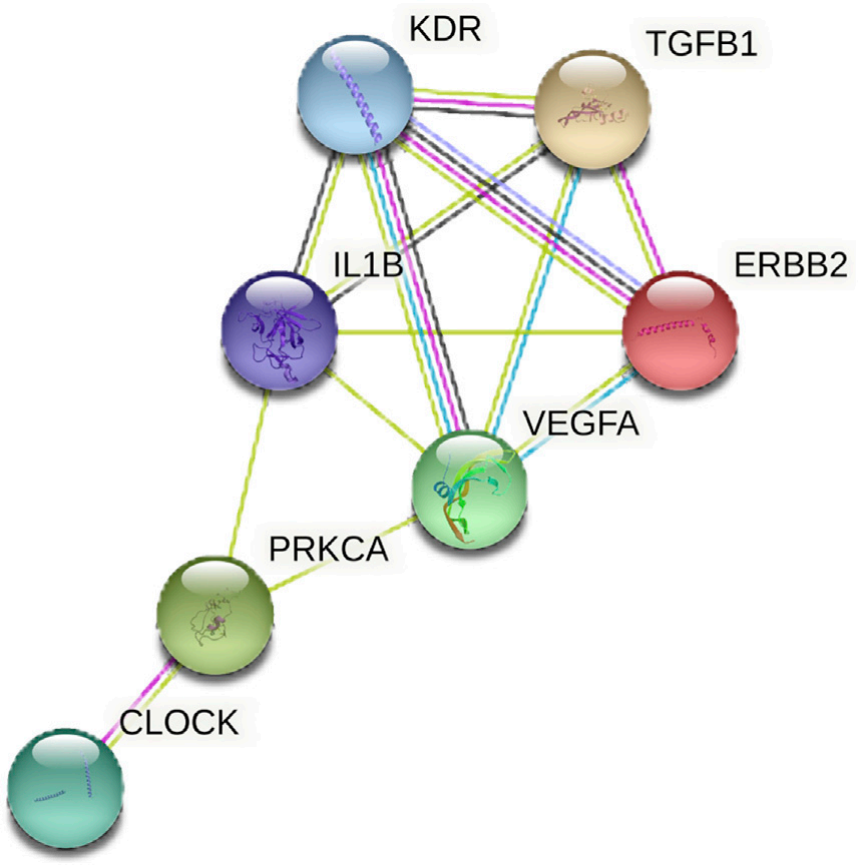
PPI network diagram of CLOCK and intersection genes in MAPK signaling pathways. The network shows predicted protein-protein interactions between CLOCK and key target genes (PRKCA, IL1B, ERBB2, KDR, VEGFA, TGFB1) involved in the MAPK signaling pathway. Lines indicate different types of evidence for the interaction.

In rats subjected to continuous light exposure, BAX protein expression increased, Bcl-2 expression decreased, and the apoptosis rate of ovarian cells increased significantly (Figure 7). Post-ZYBYF treatment, BAX expression decreased, Bcl-2 expression increased, and the apoptosis rate of ovarian cells decreased, indicating that ZYBYF has a protective effect against apoptosis in the context of CRD-induced PCOS.

## Discussion

The pathogenesis and treatment of PCOS remain inadequately understood (Dehghani et al., 2020). Circadian rhythm disruption may play a role. This study aimed to explore the lncRNA TCONS_00265853-miR-421-5p-*CLOCK* axis in PCOS with circadian rhythm disruption and investigate the therapeutic mechanism of ZYBYF. Results indicate that PRKCA is downregulated by circadian rhythm disruption, potentially due to decreased lncRNA TCONS_00265853, leading to increased miR-421-5p, decreased *CLOCK* expression, and activation of the MAPK signaling pathway. These molecular changes may induce or exacerbate PCOS. Notably, ZYBYF treatment appears to reverse these molecular events, suggesting its potential in managing PCOS with circadian rhythm disruption.

Circadian rhythm disruption is strongly associated with PCOS. It increases the risk of metabolic diseases such as obesity and diabetes (Shetty et al., 2018), which are often comorbid with PCOS (Sam, 2007). Additionally, studies have reported that circadian rhythm disruption elevates the risk of hyperandrogenemia and PCOS (Li et al., 2020). Recent findings highlight a positive correlation between PCOS incidence and night shifts or shift frequency (Wang F. et al., 2021). Furthermore, clock genes *BMAL1* and *CLOCK* are significantly lower in PCOS patients compared to non-PCOS individuals (Johnson et al., 2022). A case-control study involving 268 women found that idiopathic recurrent spontaneous abortion was linked to variants in two regions of the *CLOCK* gene (Hodzic et al., 2018). Animal studies demonstrated that *CLOCK* knockout results in reduced fertility and increased abortion rates in mice, implying that reduced *CLOCK* expression impairs reproductive functions (Li et al., 2015). These findings underscore the close relationship between CLOCK and female reproductive endocrinology, with its abnormal expression detrimental to reproductive function.

Previous studies have shown that ZYBYF improves hormone levels in PCOS (Ren and Tan, 2006). By integrating data on PCOS, CRD, and ZYBYF, 25 intersecting genes were identified. Both GO and KEGG enrichment analyses consistently emphasized the significance of the MAPK pathway. MAPKs are signal transduction enzymes that regulate gene expression, cell proliferation, and apoptosis (Yue and Lopez, 2020). The MAPK signaling pathway is implicated in both PCOS and circadian rhythm regulation (Rawashdeh et al., 2018). In this study, protein interaction network analysis suggested that CLOCK modulates the expression of other genes within the MAPK signaling pathway by interacting with PRKCA. This aligns with our experimental results showing that CRD downregulated *Prkca* expression while upregulating other MAPK-related genes, a trend reversed by ZYBYF treatment.

Ovarian granulosa cell apoptosis is closely associated with PCOS development (Huang et al., 2021). Increased granulosa cell apoptosis is a critical factor in PCOS pathogenesis.

The study also identified six key genes (*PRKCA, IL1B, ERBB2, KDR, VEGFA*, and *TGFB1*) as critical targets. PRKCA, a member of the PKC family, affects various physiological processes through the MAPK signaling pathway (Wang M. et al., 2021). The dysregulation of *IL1B* (de Alencar et al., 2016), *VEGFA* (Abd El Aal et al., 2005), *KDR* (Duan et al., 2017), *ERBB2* (Day et al., 2015), and *TGF-β1* (Yang et al., 2015) has been previously linked to PCOS, corroborating their identification as key targets in our model. ZYBYF’s ability to normalize the expression of these genes suggests a multi-target mechanism of action.

These findings suggest that ZYBYF exerts inhibitory effects on the hyperactive MAPK signaling pathway through the lncRNA TCONS_00265853-miR-421-5p-*CLOCK* axis, thereby reducing ovarian cell apoptosis and improving reproductive endocrine status in rat models. Therefore, individuals at high risk of PCOS and those already diagnosed with PCOS should avoid late sleep and night work to prevent circadian rhythm disruption. Additionally, TCM treatment, particularly ZYBYF, may assist women seeking to conceive by restoring menstrual regularity and enhancing fertility.

This study has limitations. First, while our results strongly suggest a regulatory relationship within the TCONS_00265853-miR-421-5p-CLOCK axis, we did not perform functional perturbation experiments, such as knockdown or overexpression assays, to definitively establish causality. Therefore, the mechanistic links proposed remain correlational and require further validation. Second, our validation was conducted exclusively through *in vivo* experiments; future *in vitro* studies using granulosa cells will be necessary to dissect the molecular mechanisms of both PCOS pathogenesis and ZYBYF’s action.

## Conclusion

The results demonstrate that circadian rhythm disruption promotes PCOS through the lncRNA TCONS_00265853-miR-421-5p-*CLOCK* axis, causing PRKCA downregulation, MAPK pathway overactivation, and excessive ovarian apoptosis. ZYBYF effectively reverses this pathogenic cascade, representing a promising therapeutic approach for circadian-related PCOS. Future research should focus on translational validation and comparative effectiveness studies.

## Data Availability

The original contributions presented in the study are included in the article/supplementary material, further inquiries can be directed to the corresponding authors.
